# Bioinformatics and evolutionary insight on the spike glycoprotein gene of QX-like and Massachusetts strains of infectious bronchitis virus

**DOI:** 10.1186/1743-422X-9-211

**Published:** 2012-09-19

**Authors:** Shahid Hussain Abro, Karin Ullman, Sándor Belák, Claudia Baule

**Affiliations:** 1 Department of Biomedical Sciences and Veterinary Public Health, Section of Virology, The Swedish University of Agricultural Sciences, Ulls Väg 2B, SE-751 89, Uppsala, Sweden; 2 Department of Virology, Immunobiology and Parasitology, The National Veterinary Institute, Ulls Väg 2B, SE-751 89, Uppsala, Sweden

**Keywords:** Infectious bronchitis virus, Spike glycoprotein, Bioinformatics, Coronavirus, Evolution

## Abstract

**Background:**

Infectious bronchitis virus (IBV) is a Gammacoronavirus of the family C*oronaviridae* and is a causative agent of an economically important disease in poultry. The spike glycoprotein of IBV is essential for host cell attachment, neutralization, and is involved in the induction of protective immunity. Previously obtained sequence data of the spike gene of IBV QX-like and Massachusetts strains were subjected to bioinformatics analysis.

**Findings:**

On analysis of potential phosphorylation sites, the Ser542 and Ser563 sites were not present in Massachusetts strains, while QX-like isolates did not have the Ser534 site. Massachusetts and QX-like strains showed different cleavage site motifs. The N-glycosylation sites ASN-XAA-SER/THR-55, 147, 200 and 545 were additionally present in QX-like strains. The leucine-rich repeat regions in Massachusetts strains consisted of stretches of 63 to 69 amino acids, while in the QX-like strains they contained 59 amino acids in length. An additional palmitoylation site was observed in CK/SWE/082066/2010 a QX-like strain. Primary structure data showed difference in the physical properties and hydrophobic nature of both genotypes. The comparison of secondary structures revealed no new structural domains in the genotypic variants. The phylogenetic analyses based on avian and mammalian coronaviruses showed the analysed IBV as closely related to turkey coronaviruses and distantly related to thrush and munia coronaviruses.

**Conclusion:**

The study demonstrated that spike glycoprotein of the Massachusetts and the QX-like variants of IBV are molecularly distinct and that this may reflect in differences in the behavior of these viruses in vivo.

## Background

Infectious bronchitis virus (IBV) causes avian infectious bronchitis, a highly contagious disease that affects poultry and produces severe economic losses worldwide. IBV belongs to the order of Nidovirales, family *Coronaviridae* and to genus of Gamma-coronavirus group 3 [1]. The genome is positive-sense single stranded RNA of about 27.6 kb and contains 5′ and 3′ untranslated regions [2-4].

The Massachusetts strain was initially isolated in 1940 in USA and clinically characterized as causing respiratory signs and decrease in egg production. It is widely distributed and predominant in many countries around the world [5]. The Massachusetts strain can infect different organs and some of the isolates could be recovered from gastrointestinal tract especially from the cecal tonsils [6,7]. First IBV QX infections characterized by swelling of stomach in chicken flocks were reported in Qingdao China in 1996 [8]. Following the years the IBV QX viral infections was associated to either proventriculitis or renal infections in IBV vaccinated flocks in China [9,10]. The decreases in egg production and false layers in mature hens in chicken flocks were also reported with QX infections [10,11]. Later on Chinese QX of IBV was isolated from a backyard flock in Russia where the prevalence of Chinese QX-like genotype was common [12,13]. Subsequently there have been increasing reports of QX-like cases in Europe, where the involved virus bearded sequence similarities to QX-like viruses, such as the cases in Belgium, Denmark, France, Hungry, Germany, The Netherlands, Poland, Russia, Slovenia, Spain, Sweden, and UK [7,14-19]. These IBV viruses were termed as European QX-like viruses symptoms associated with bad egg quality, false layers in mature hens [2].

The spike glycoprotein of the virus is translated as a pre-cursor protein (S_O_) that is later cleaved into the N-terminal S1 and C-terminal S2 glycopolypeptides. The spike gene is highly variable, especially the S1 part, due to insertions, deletions, substitutions and recombination events [14,20,21]. The S1 part of the spike glycoprotein contains serotype specific virus neutralizing epitopes and is responsible for the hemagglutinating activity and for infectivity [22]. Due to this variability in nucleotide sequences, the cross protection between serotypes is low. Changes as little as 5% in the S1 sub-unit have been able to alter the protection ability of a vaccine [23]. The spike glycoprotein cleavage site motif has been described in bovine coronavirus, turkey coronavirus and human coronavirus, however, it is not found in all coronaviruses [24]. The cleavage recognition site in the spike glycoprotein of IBV consists of five basic amino acids, which is cleaved by host cell serine proteases [25]. Serine proteases are hydrolases that cleave peptide bonds [26,27]. The cleavage recognition site sequence is not involved in pathogenicity because attenuated and pathogenic strains (same virus) possess identical cleavage recognition site sequences [26]. The spike glycoprotein of IBV is involved in induction of protective immunity, neutralization and attachment to the host cell [28,29]. It contains crucial virus neutralizing epitopes and serotypic specific sequences that are involved in, induction of protective immunity, tissue tropism and attachment into host cells [22,25,30-32].

Palmitoylation is essential for the screening, localization and trafficking at sub-cellular level, protein-protein interactions, and to pre-determine functional properties of proteins [33,34]. N-glycosylation properties of the glycoprotein were shown to be involved in changes of virulence and cellular tropism in lactate dehydrogenase-elevating virus [35]. Protein phosphorylation has key role in regulation of physiological functions in the cells of prokaryotes and eukaryotes [36]. Leucine-rich repeat (LRR) is a structural motif of the protein like a alpha/beta horseshoe fold which is relevant for protein-protein interactions [37,38]. In this study, palmitoylation, leucine-rich repeat and N-glycosylation characteristics of IBV Massachusetts and QX-like strains were evaluated using bioinformatics tools in order to determine the extent of difference in structural and functional features between the strains.

The objective of the present study was to analyze the molecular characteristics of selected isolates belonging to classical (Massachusetts) and emerging (QX-like) genotypes of IBV to indentify differences in potential functional motifs of the spike glycoprotein, predicted by bioinformatics analysis. In addition an evolution analysis with regards to avian and mammalian coronavirus was performed.

## Results

### Prediction of N-glycosylation

The loss or acquisitions of N-glycosylation sites in the spike protein were predicted. Thirty N-glycosylation sites were found in the Massachusetts strains, while 35 N-glycosylation sites were present in the QX-like strains. The N-glycosylation sites ASN-XAA-SER/THR-55, 147, 200 and 545 were additionally present in spike gene of QX-like strains. The results revealed that most of the N-glycosylation sites were conserved within genotype in the analyzed strains, except in strain CK/SWE/082066/10 that had lost ASN-XAA-SER/THR-533.

### Analysis of potential phosphorylation sites

The analyses revealed that there were different in number and location of potential phosphorlated peptides in the spike glycoprotein. In Massachusetts isolates phosphorylation sites at position Ser54, Ser79, Ser312, Ser404, Ser534, Ser618, Ser685, Ser908 and Ser1061 were found to be conserved. While two isolates CK/SWE/242/95 and CK/SWE/748/95 that did not contain phosphorylation sites at position Ser542 and Ser563. In contrast, they contained additional potential phosphorylation sites at Ser177 and Ser878. QX-like strains did not contain a potential phosphorylation site at position Ser534 and acquired a new potential phosphorylation site at position Ser513.

### Proteolytic cleavage sites in the spike glycoprotein

The cleavage site motifs of analyzed strains are presented in Table 1. Massachusetts and QX-like strains showed a different cleavage site motif. From the analyzed strains of both genotypes, six strains have shown the cleavage site at amino acid position 537 arginine, while two strains CK/SWE/242/95 and CK/SWE/748/95 presented cleavage site at histidine.

**Table 1:** Cleavge site motif in Swedish IBV strains

**Strian**	**Type**	**Cleavage site sequence motif**
CK/SWE/242/95	Massachusetts	^535^Aln-Gly-His-Ser-Ile^539^
CK/SWE/748/95	Massachusetts	^535^ Aln-Gly-His-Ser-Ile^539^
CK/SWE/423/97	Massachusetts	^535^ Leu-Arg-Arg-Ser-Ile^539^
CK/SWE/1096/97	Massachusetts	^535^ Leu-Arg-Arg-Ser-Ile^539^
CK/SWE/062545/09	QX-like	^535^ Ser-His-Arg-Thr-Arg^539^
CK/SWE/062561/09	QX-like	^535^ Ser-His-Arg-Thr-Arg^539^
CK/SWE/079692/10	QX-like	^535^ Ser-Gln-Arg-Arg-Arg^539^
CK/SWE/082066/10	QX-like	^535^ Ser-Gln-Arg-Arg-Arg^539^

Cleavage site at amino acid position 537 =

Amino acid abbreviations: Aln = alanine, Gly = glycine, His = histidine, Ser = serine.

Ile = Isoleucine, Thr = threonine, Arg = arginine, Gln = glutamine.

### Determination of leucine-rich repeat regions

Leucine-rich repeat (LRR) regions were observed in the spike glycoprotein consisting of 59 to 69 amino acids in length. In isolate CK/SWE/748/95 two LRR regions were found, starting at position at 771 (ATQLQARNNAL) and 1147 (LEKLSILKTYI), respectively. The LRR regions in Massachusetts strains consisted of stretches of 63 to 69 amino acids, while in the QX-like strains they contained 59 amino acids in length.

### Prediction of palmitoylation sites

The isolates CK/SWE/748/95, CK/SWE/1096/97 and CK/SWE/062545/09 contained 10 palmitoylation sites in the spike glycoprotein of the virus. The isolate CK/SWE/082066/2010 contained additional palmitoylation site 1130 (CGGCFGI) in comparison to the all other analyzed isolates.

### Primary structure of the spike glycoprotein

The data regarding the primary structures are presented in (Additional file 1: Table S1). The strains of Massachusetts group were found to have differences of approximately 0.3-1.2 KDa in their molecular weights, while QX-like strains possessed similar molecular weight. The isoelectric points (pI) 5.63-6.72 of the Massachusetts strains were comparatively higher than pI 5.73 of QX-like strains. There were 82-87 positively charged residues (Arg + Lys) in the QX-like strains, 2-4 residues more than Massachusetts strains. The computed instability index and aliphatic index (AI) showed variable values (83.79-85.91) in all analyzed strains. The grand hydropathicity (GRAVY) was found to have negative values in all strains. The primary structure data showed difference in the hydrophobic nature of both genotypes. The primary structures of the spike glycoprotein were found to be relatively variable in the strains.

### Prediction of secondary structures of spike glycoproteins

The data regarding the secondary structures are presented in (Additional file 2: Table S2). All the analyzed isolates contained 18 to 19 alpha helices, 47-58 beta sheets and 63-73 residual coils. In the case of alpha helices and beta sheets, there was additionally one alpha helix and 11 beta strands in Massachusetts strains than in QX-like strains.

### Phylogenetic analysis

**Figure 1:**
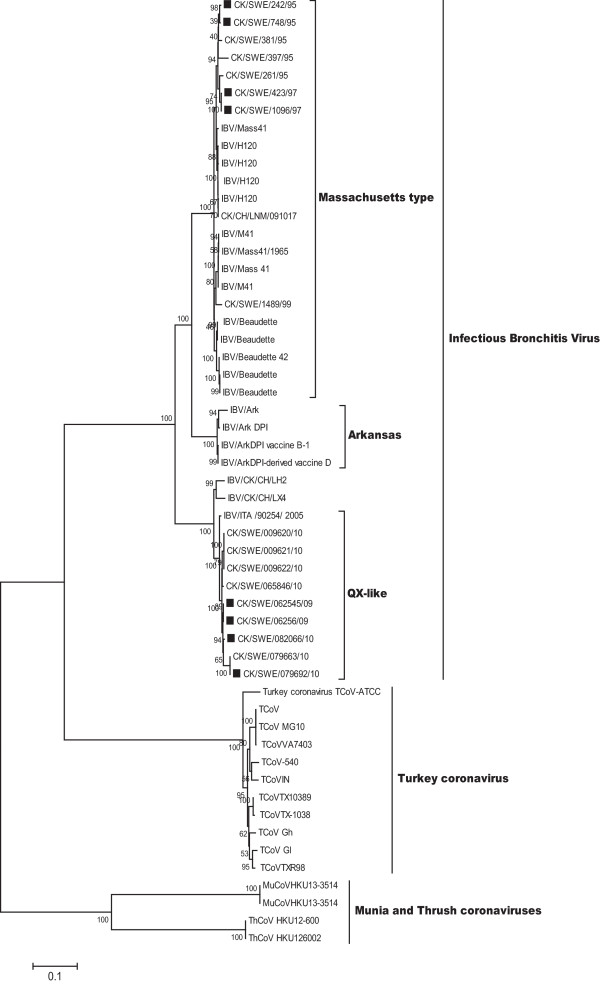
**Phylogenetic analysis based on complete spike gene of avian coronaviruses showing the relationship between IBV and avian coronaviruses.** The isolates analysed in this study are indicated by square.

The phylogenetic analysis of the complete spike gene based on avian coronaviruses revealed the sequences of the analysed strains segregated into three main groups (Figure 1). Group I was composed of IBV sequences that clustered together with those of Massachusetts, Arkansas and QX-like genotypes, as previously described by Abro et al. (2012) [14]. The definition of those genotypes is supported by high bootstrap values of 100%. Group II consisted of turkey coronaviruses branching closely to the IBV. Group III consisted of sequences of munia and thrush coronaviruses, branching into two clusters: one comprising munia coronaviruses and the other thrush coronaviruses. The high bootstrap values (100%) indicate a true distance between these clusters and that they are likely species-specific.

**Figure 2:**
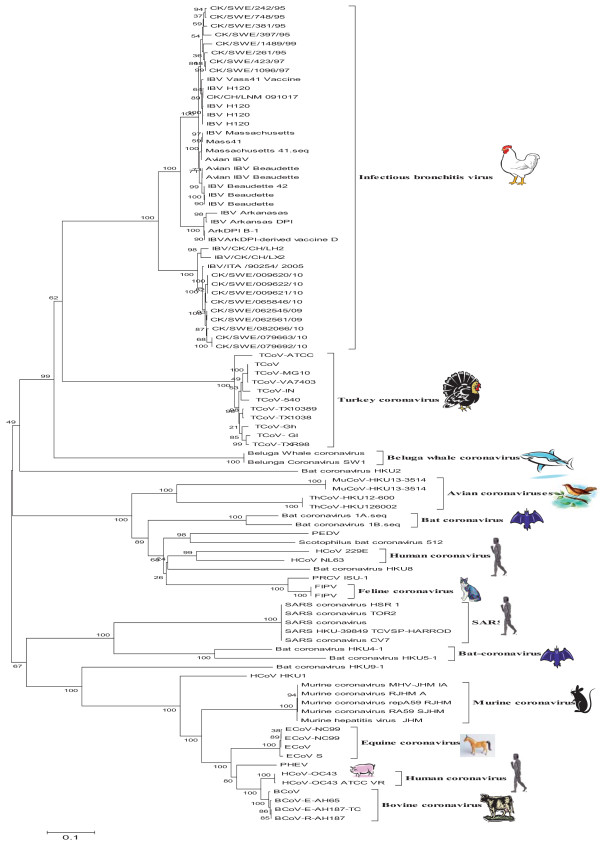
Phylogenetic analysis based on complete spike gene of avian and mammalian coronaviruses showing the relationship between coronaviruses in different host species.

The phylogenetic analysis based on the spike glycoprotein of avian and mammalian coronaviruses strains has shown as the following discrimination (Figure 2): a) Sequences of IBV and turkey coronaviruses making the majority of avian coronaviruses. The sequences of Beluga whale coronaviruses and one bat-coronavirus HKU2 were distantly related to this branch of avian sequences, altogather composing the gama-coronaviruses. b) A set of sequences comprising separate clusters of avian, bat, human and feline coronaviruses, thus including both avian and mammalian coronaviruses. c) A set of strictly mammalian coronaviruses, branching into the SARS, human coronavirus HKU1, bat coronaviruses and a cluster majorly of animal coronaviruses comprising murine, equine, porcine and bovine coronaviruses. The sequence of human coronavirus OC43 was found in this cluster, curiously identical to a coronavirus identified in pigs and closely related to bovine coronaviruses. Porcine hemagglutinating encephalomyelitis virus occupied a distinct place between the human and equine coronaviruses clusters.

## Discussion

A genetic analysis of the complete spike glycoprotein of selected isolates of Massachustes and QX-like strains was carried out in order to predict molecular features by application of molecular and *In silico* technologies.

In the isolate CK/SWE/082066/10, amino acid phenylalanine substituted asparagine resulting in the loss of a potential N-glycosylation site at position 533. The five additional N-glycosylation sites found in QX strains may influence cellular tropism. The variation in N-glycosylation sites may affect survival and transnsmission of the virus. The small changes can lead to disturbance in folding and conformation of the molecule. The variations in N-glycosylation sites may affect the interaction with receptors and render a virus more susceptible to host innate immune system and lower recognition by antibody, hence affecting the virus replication and infectivity [39-42]. In a virus belonging to the *Coronaviridae*, lactate dehydrogenase elevating virus (LDEV), the loss or acquisition of N-glycosylation sites in the protein has been reported to determine changes of virulence and cellular tropism [35].

The presence of serine, threonine and tyrosine residues, with the right context for potential phosphorylation was investigated in the spike glycoprotein. The results have shown that the spike glycoprotein of IBV genotypes contained serine and thereonine peptides with potential for phosphorylation. The substitution of a serine by amino acid alanine at positions 563 caused loss of a potential phosphorylated peptide in strains CK/SWE/242/95 and CK/SWE/748/95. Wilbur et al. (1986) reported that N protein is the only structural protein that contained phosphorylation activity in coronaviruses [43]. In this context we have observed potential phosphorylated peptides in the spike glycoprotein of IBV; however, this seems to be of no consequence for phosphorylation, since this property has not been identified in the spike which is well established as a glycoprotein.

The difference in cleavage site motifs of the spike glycoprotein of both genotypes, seem to indicate a correlation with geographical distribution of strains. It has been reported that serotypically and genotypically different IBV contain distinct cleavage site in the spike protein. However, the cleavage site sequence did not correlate with pathogenicity by comparing cleavage recognition sites in pathogenic and attenuated strains of IBV [26]. The cleavage site motifs determined in Massachusetts and QX-like strains in present study are different from previously reported by Jackwood et al. (2001) [26]. The replacement of arginine with histidine at amino acid position 537 in CK/SWE/748/95 and CK/SWE/748/95 may have influence antibody binding epitope. However, the functional meaning of this difference needs to be evaluated in proper biological assays. It has been reported that an IBV monoclonal antibody neutralization-resistant mutant (NR 18) had shown substitution of amino acid isoluecine for arginine in cleavage site motif which prevented cleavage and alter the conformation dependent monoclonal antibody binding epitope [26]. In orthomyxoviruses and paramyxoviruses more basic residues around the cleavage recognition site of the envelope glycoproteins enhance the cleavability by ubiquitous host cell proteases, a characteristic which is associated with host cell range and increased virulence [44,45]. However, the relevance of cleavage site to virulence was not observed in case of IBV spike glycoprotein [26].

The data regarding leucine-rich repeat regions of isolate CK/SWE/748/95 demonstrated that two LRR regions may result in strong folding capacity in the spike glycoprotein. It has been reported that leucine-rich repeat (LRR) is a structural motif of protein like a alpha/beta horseshoe fold and is responsible for protein-protein interactions [37,38].

An additional palmitoylation site was found only in isolate CK/SWE/082066/10, indicating that it is strain specific. However, experimental studies are required to identify the effect of additional palmitoylation in the virus biology. In influenza virus, palmitoylation of the cytoplasmic domain of the hemagglutinin has been shown to increase its association with lipid mass [46]. The deletion of palmitoylation sites in the hemagglutinin domain reduces hemagglutinin incorporation into the virion, thereby affecting the cellular trafficking [47]. It has been reported that removal of palmitoylation sites in HIV-1 virus effected in the formation of virus with low levels of glycoproteins incorporation and reduced infectivity [48]. Shen et al. (2009) reported attenuation of virus replication occurring due to mutations in the palmitoylation site in the non-structural protein nsp1 of Semliki Forest virus [49].

The predicted primary structures of the spike glycoprotein of both genotypic variants have shown a variable trend in physical properties such as isoelectric point, molecular weights and grand hydropathicity index. Grand average of hydropathicity index results suggested a hydrophobic nature of the spike glycoprotein in all variants. Cavanagh et al. (1986) reported that S2 sub-unit in N-terminal side of the membrane spanning hydrophobic domain of spike glycoprotein of IBV is susceptible to hydrolysis by many proteases [32].

The secondary structures prediction revealed that structures of the spike glycoprotein were found variable in both genotypic variants. It was also observed that there were no new structural domains and that no evolution has occurred in the structure of both genotypic variants. However, secondary structural variation in beta strands and residual coils have been observed between both genotypic strains.

A number of phylogenetic studies performed on the spike gene were mostly based on the partial sequences of S1 and N gene. Phylogenetic analyses based on the complete spike glycoprotein of avian coronaviruses demonstrated that IBV strains showed higher relatedness to turkey coronaviruses in comparison to other avian coronaviruses (Figure 1). This could be due to co-habitation of their host species. The appearance of a bat coronavirus branch close to these gammacoronaviruses suggests a possible common origin during earlier evolution of these viruses. The diversity of these viruses could have arisen from strong selective pressure resulting from mutations and recombinations that has been shown to occur in spike gene [14,50,51].

Phylogenetic analysis based on spike glycoprotein of avian coronaviruses and mammalian coronaviruses had revealed interesting trends. The mammalian beluga whale coronaviruses branched closer to the avian coronaviruses and two avian thrush and munia coronaviruses were related to the mammalian coronaviruses suggesting interspecies transmission. Furthermore, findings of the present study suggest that genetic diversity and molecular variation in sequences of spike glycoprotein of viruses made feasible the adaptation to new host species. Previously it has been reported that the RNA-dependant RNA polymerase gene and the whole genome of the beluga whale coronavirus was distantly related to IBV [52,53]. Also a shift of tissue tropism in the virus has been reported [54,55] resulting in infection of a wide range of avian host species especially those reared close to domestic fowl. For example, IBV cases were found in guinea fowl (Numida meleagris), Chinese peafowl (Pavo), teal (Anas) and partridge (Alectoris) [56].

The higher diversity in the avian, humans and bat-coronaviruses are due to continuous mutation, which may determine differences in tissue tropism. Previously it has been reported that diversity of coronaviruses due to a) infidelity of RNA-dependant RNA polymerase which lead to incorporation of nucleotide errors during replication [34,57]; b) random template switching during RNA replication (copy choice mechanism) high rate of homologous RNA recombination events [4,58]; c) extra plasticity nature in adjusting and modifying genes [59]. These factors were responsible for the generation and diversification of variants and adaption to new hosts and ecological niches in the coronaviruses [53,59].

## Conclusions

In silico predictions confirm previous data on phylogenetic analysis, showing that the spike glycoprotein of the Massachusetts and QX genotypes are molecularly different. Since the spike glycoprotein has important role in cell attachment, receptor recognition, the genetic diversity seen in this protein of IBV, avian, human, bat, and other animal coronaviruses may bear relation with host specificity. The identification of these characteristics will be foundation for the better understanding of epidemics, molecular mechanisms of infections, evolution and basis for development of effective vaccines. Bioinformatics approach is easy to perform, cheap and suitable to obtain predictions about functional motifs in a genome. However, the predicted findings require validation through proper laboratory experimentation.

## Methods

### Sequence data set

The sequence data set generated as previously described by Abro et al. (2012) [14] was used for the analysis (Additional file 3: Table S3).

### Bioinformatics analysis

N-glycosylation sites were predicted by services available on http://www.cbs.dtu.dk/services/NetNGlyc. The potential phosphorylation sites were determined by using website http://www.cbs.dtu.dk/services/NetPhos. Leucine-rich repeat regions were determined by LRRfinder available at http://www.lrrfinder.com/result.php. Antigenic determinants were predicted with the maximum score with the high confidence for positive determinant motif in the amino acid sequence with the help of server http://mobyle.pasteur.fr. The primary structures of the spike glycoprotein were predicted by http://expasy.org/tools. Palmitoylation sites were predicted with the medium threshold frequency by using services at http://csspalm.biocuckoo.org/prediction.php. The secondary structures (alpha helices, beta strands and random coils) of the protein were predicted by using bioinformatics tools available on website http://npsa-pbil.ibcp.fr. The method of GOR4 http://npsa-pbil.ibcp.fr/cgi-bin/npsa_automat.pl?page=npsa_gor4.html was used to identify the alpha helices, beta strands and coil residues. At least minimum three or more turns were taken in account for one helix, strand or coil in the structure of spike glycoprotein.

### Phylogenetic analysis

The comprehensive phylogenetic study was carried out based on the complete spike gene of avian and mammalian coronaviruses available in GenBank. The sequences included IBV Massachusetts type, M41, H120, Baudette, Arkansas, QX like strains and other sequences of Turkey coronavirus (TCoV), thrush coronavirus (ThCoV), munia coronavirus (MuCoV), human coronavirus (HCoV), SARS (Severe acute respiratory syndrome) coronavirus, bovine coronavirus (BCoV), equine coronavirus (ECoV), mouse hepatitis virus (MHV), murine coronavirus (MurCoV), feline coronavirus (FCoV), porcine epidemic diarrhea virus (PEDV), porcine respiratory coronavirus (PRCV), porcine hamagglutinating encephalalomylitis virus (PHEV), bat coronavirus and beluga whale coronavirus sequences (Additional file 3: Table S3; Additional file 4: Table S4). Multiple sequence alignment of complete spike gene was performed with CLUSTAL W [60]. All the gaps in the sequences created by insertions were deleted by using software Mega4 version 4 [61]. Phylogenetic trees were constructed by the Neighbour-joining method with the nucleotide substitution model of Kimura-2 parameter model. The tree reliability/robuststness of the hypothesis were evaluated by bootstrap of 1000 replicates. The data set was kept similar for the analysis. The Bayesian analyses were performed by using the software MrBayes 3.1 [62]. The model was set for the substitution rates lset nst = 6, rates of variation were performed by gamma distribution (rates = invgamma) posterior probability distribution ensured and 2000000 generation were run and standard deviation of split frequencies at .01. The output of the substitution model parameters were summarized with sump burnin = 250. The tree topology was prepared in the FigTree_v1.3.1. The trees were generated with various methods and the consensus tree was taken into account in this study.

## Competing interests

The authors have declared that they have no competing interests.

## Authors’ contributions

Study concepts and design: SHA, CB Performance of experiments: SHA, KU Analysis of the data: SHA, CB Writing of the manuscript: SHA, KU, SB, CB. The authors have read and approved the final manuscript.

## Supplementary Material

Additional file 1: Table S1 Predicted primary structures of spike glycoprotein. Click here for file

Additional file 2: Table S2 Predicted secondary structures of spike glycoprotein. Click here for file

Additional file 3: Table S3 List of selected Swedish IBV strains and reference strains of avian coronaviruses of which sequences were downloaded from GenBank for the phylogenetic analysis in this study. The IBV strains analyzed in this study are indicated by star *. Click here for file

Additional file 4: Table S4 List of reference strains of mammalian coronavirus sequences were downloaded from GenBank for the phylogenetic analysis in this study. Click here for file
