# Supplementary material for: Bioinformatics and evolutionary insight on the spike glycoprotein gene of QX-like and Massachusetts strains of infectious bronchitis virus
Source: Virol J. 2012 Sep 19;9:211. doi: 10.1186/1743-422X-9-211 (PMC3502414; doi:10.1186/1743-422X-9-211)
Supplement: Additional file 1: Table S1 — Predicted primary structures of spike glycoprotein. [file 1743-422X-9-211-S1.docx]

Table S1. Predicted primary structures of spike glycoprotein

| Isolate | M. wt. | pI | -ve Res.  (Asp+Glu) | +ve Res.  (Arg+Lys) | II | AI | GRAVY |
| --- | --- | --- | --- | --- | --- | --- | --- |
| CK/SWE/242/95 | 128096.2 | 5.98 | 85 | 75 | 34.87 | 85.91 | - 0.006 |
| CK/SWE/478/95 | 127955.0 | 6.13 | 84 | 76 | 33.99 | 84.96 | - 0.008 |
| CK/SWE/423/97 | 128207.5 | 6.72 | 83 | 79 | 37.90 | 84.13 | - 0.032 |
| CK/SWE/1096/97 | 128180.5 | 6.72 | 83 | 79 | 37.90 | 84.04 | - 0.030 |
| CK/SWE/062545/09 | 128587.9 | 5.81 | 91 | 82 | 33.82 | 84.22 | - 0.017 |
| CK/SWE/062561/09 | 1285887.9 | 5.81 | 91 | 82 | 33.83 | 84.22 | - 0.017 |
| CK/SWE/079692/10 | 128835.4 | 5.63 | 90 | 86 | 35.34 | 83.79 | - 0.028 |
| CK/SWE/082066/10 | 1288690 | 5.90 | 92 | 84 | 35.01 | 83.89 | - 0.027 |
|  |  |  |  |  |  |  |  |

M. wt. = Molecular weight

pI = Isoelectric point

-ve Res.= Negatively charged residues

+ve Res.= Positively charged residues

II = Instability index

AI = Aliphatic index

GRAVY = Grand average of hydropathicity
