# Supplementary material for: Bioinformatics and evolutionary insight on the spike glycoprotein gene of QX-like and Massachusetts strains of infectious bronchitis virus
Source: Virol J. 2012 Sep 19;9:211. doi: 10.1186/1743-422X-9-211 (PMC3502414; doi:10.1186/1743-422X-9-211)
Supplement: Additional file 2: Table S2 — Predicted secondary structures of spike glycoprotein. [file 1743-422X-9-211-S2.docx]

Table S2. Predicted secondary structures of spike glycoprotein

| Isolate | Alpha helices | Beta sheets | Residual coils |
| --- | --- | --- | --- |
| CK/SWE/242/95 | 19 | 58 | 68 |
| CK/SWE/478/95 | 19 | 58 | 73 |
| CK/SWE/423/97 | 19 | 57 | 63 |
| CK/SWE/1096/97 | 19 | 56 | 65 |
| CK/SWE/062545/09 | 18 | 47 | 66 |
| CK/SWE/062561/09 | 18 | 46 | 73 |
| CK/SWE/079692/10 | 18 | 46 | 72 |
| CK/SWE/082066/10 | 18 | 45 | 68 |
|  |  |  |  |
