# Supplementary material for: Bioinformatics and evolutionary insight on the spike glycoprotein gene of QX-like and Massachusetts strains of infectious bronchitis virus
Source: Virol J. 2012 Sep 19;9:211. doi: 10.1186/1743-422X-9-211 (PMC3502414; doi:10.1186/1743-422X-9-211)
Supplement: Additional file 3: Table S3 — List of selected Swedish IBV strains and reference strains of avian coronaviruses of which sequences were downloaded from GenBank for the phylogenetic analysis in this study. The IBV strains analyzed in this study are indicated by star *. [file 1743-422X-9-211-S3.docx]

Table S3. List of selected Swedish IBV strains and reference strains of avian coronaviruses of which sequences were downloaded from GenBank for the phylogenetic analysis in this study.

| Strian | Type | Country | NCBI Accession number |
| --- | --- | --- | --- |
| CK/SWE/242/95* | Massachusetts | Sweden | JN022536 |
| CK/SWE/261/95 | Massachusetts | Sweden | JN022537 |
| CK/SWE/381/95 | Massachusetts | Sweden | JN022538 |
| CK/SWE/397/95 | Massachusetts | Sweden | JN022539 |
| CK/SWE/423/97* | Massachusetts | Sweden | JN022540 |
| CK/SWE/748/95* | Massachusetts | Sweden | JN022541 |
| CK/SWE/1096/97* | Massachusetts | Sweden | JN022542 |
| CK/SWE/1489/99 | Massachusetts | Sweden | JN022543 |
| CK/SWE/6904/99 | Massachusetts | Sweden | JN022544 |
| CK/SWE/09620/10 | QX-like | Sweden | JN022545 |
| CK/SWE/09621/10 | QX-like | Sweden | JN022546 |
| CK/SWE/09622/10 | QX-like | Sweden | JN022547 |
| CK/SWE/062545/09* | QX-like | Sweden | JN022548 |
| CK/SWE/062561/09* | QX-like | Sweden | JN022549 |
| CK/SWE/065846/10 | QX | Sweden | JN022550 |
| CK/SWE/079663/10 | QX-like | Sweden | JN022551 |
| CK/SWE/079692/10* | QX-like | Sweden | JN022552 |
| CK/SWE/082066/10* | QX-like | Sweden | JN022553 |
| CK/SWE/900419/97 | Massachusetts | Sweden | JN022554 |
| CK/SWE/A889/99 | Massachusetts | Sweden | JN022555 |
| LX4 | QX-like | China | AY189157 |
| LH2 | QX-like | China | AY180958 |
| ArkDPI vaccine B-1 | Arkansass |  | EU359645 |
| Avian IBV Beaudette | Beaudette |  | DQ001335 |
| H120 | H120 | Netherlands | GU393335 |
| H120 | H120 | Taiwan | EU822341 |
| H120 | H120 | Netherlands | FJ888351 |
| CK/CH/LNM/091017 |  | China | JF330899 |
| IBV H120 | H120 | China | FJ807652 |
| IBV Beaudette | Beaudette | Singapore | DQ001340 |
| IBV Beaudette | Beaudette | Singapore | DQ001335 |
| IBV Beaudette | Beaudette | Singapore | DQ001341 |
| IBV isolate Beaudette 42 | Beaudette | USA | DQ830981 |
| IBV Ark Vaccine | Arkansas | USA | GQ504721 |
| IBV Ark DPI | Arkansas | USA | GQ504720 |
| IBVArkDPI-derived vaccine D | Arkansas | USA | EU359651 |
| ArkDPI vaccine B-1 | Arkansas | USA | EU359645 |
| Massachusetts 41 | Massachusetts | USA | AY851295 |
| Mass 41 | Massachusetts | USA | DQ830980 |
| Massachusetts | Massachusetts | USA | GQ504724 |
| Mass41 1965 | Massachusetts | USA | FJ904720 |
| ITA/90254/2005 | ITA-02 |  | FN430414 |
| TCoV | Turkey coronavirus | USA | NC_010800 |
| TCoV MG10 | Turkey coronavirus | Canada | EU095850 |
| TCoV/VA-74/03 | Turkey coronavirus | USA | GQ427173 |
| TCoV/TX-1038/98 | Turkey coronavirus | USA | GU213200 |
| TCoV/TX-1038/98 | Turkey coronavirus | USA | GU213201 |
| TCoV/Gl | Turkey coronavirus | USA | AY342357 |
| TCoV/Gh | Turkey coronavirus | USA | AY342356 |
| TCoV/IN517 | Turkey coronavirus | USA | GQ427175 |
| TCoV/TX-R/98 | Turkey coronavirus | USA | GU213202 |
| TCoV-ATCC | Turkey coronavirus | USA | EU022526 |
| ThCoV/HKU12-600 | Thrush coronavirus | Hong Kong | NC_011549 |
| ThCoV/HKU12-600 | Thrush coronavirus | Hong Kong | FJ376621 |
| MuCoVHKU13-3514 | Munia coronavirus | Hong Kong | NC_011550 |
| MuCoVHKU13-3514 | Munia coronavirus | Hong Kong | FJ376622 |
