# Supplementary material for: Bioinformatics and evolutionary insight on the spike glycoprotein gene of QX-like and Massachusetts strains of infectious bronchitis virus
Source: Virol J. 2012 Sep 19;9:211. doi: 10.1186/1743-422X-9-211 (PMC3502414; doi:10.1186/1743-422X-9-211)
Supplement: Additional file 4: Table S4 — List of reference strains of mammalian coronavirus sequences were downloaded from GenBank for the phylogenetic analysis in this study. [file 1743-422X-9-211-S4.docx]

Table S4. List of reference strains of mammalian coronavirus sequences were downloaded from GenBank for the phylogenetic analysis in this study.

| Strain | Coronavirus | Country | NCBI Accession number |
| --- | --- | --- | --- |
| BCoV | Bovine coronavirus | USA | NC_003045 |
| BCoV-ENT | Bovine coronavirus | USA | AF391541 |
| BCoV E-AH187-TC | Bovine coronavirus | USA | FJ938064 |
| BCoV R-AH187 | Bovine coronavirus | USA | EF424620 |
| BCoV E-AH65 | Bovine coronavirus | USA | EF424615 |
| ECoV | Equine coronavirus | USA | NC_010327 |
| ECoV NC99 | Equine coronavirus | USA | EF446615 |
| ECoV NC99 | Equine coronavirus | USA | AY316300 |
| ECoV S | Equine coronavirus | Japan | AB555560 |
| SARS CoV | SARS coronavirus | Canada | NC_004718 |
| SARS CoV TOR2 | SARS coronavirus | Canada | AY274119 |
| SARS HKU-39849 TCVSP-HARROD-00003 | SARS coronavirus | USA | GU553365 |
| SARS CoV CV7 | SARS coronavirus | Canada | DQ898174 |
| SARS CoV HSR 1 | SARS coronavirus | Italy | AY323977 |
| Beluga Whale coronavirus | Beluga Whale coronavirus | USA | NC_010646 |
| SW1 beluga whale | Beluga Whale coronavirus | USA | EU111742 |
| FIPV | Feline coronavirus | USA | NC_002306 |
| FIPV 79-1146 | Feline coronavirus | USA | AY994055 |
| PHEV | Porcine coronavirus | Belgium | NC_007732 |
| PEDV | Porcine coronavirus |  | NC_003436 |
| TGV and PRCV ISU-1 | Porcine coronavirus | USA | DQ811787 |
| HCoV NL63 | Human coronavirus | The Netherlands | NC_005831 |
| HCoV HKU1 | Human coronavirus |  | NC_006577 |
| HCoV OC43 | Human coronavirus | Belgium | NC_005147 |
| HCoV OC43 ATCC VR-759 | Human coronavirus | USA | AY585228 |
| MHV/ JHM | Murine coronavirus |  | NC_006852 |
| MurCoV RJHM/A | Murine coronavirus | USA | FJ647219 |
| MurCoV repA59/RJHM | Murine coronavirus | USA | FJ647221 |
| MurCoV MHV-JHM.IA | Murine coronavirus | USA | FJ647226 |
| MurCoV RA59/SJHM | Murine coronavirus | USA | FJ647220 |
| Bat CoV HKU4-1 | Bat coronavirus |  | NC_009019 |
| Bat CoV HKU2/GD/430/2006 | Bat coronavirus | China | EF203064 |
| Bat CoV HKU5-1 | Bat coronavirus | China | NC_009020 |
| Bat CoV HKU8 | Bat coronavirus | Hong Kong | NC_010438 |
| Bat CoV HKU9-1 | Bat coronavirus | China | NC_009021 |
| Bat CoV1A | Bat coronavirus | Hong Kong | NC_010437 |
| Bat CoV1B | Bat coronavirus | Hong Kong | NC_010436 |
| Bat CoV512 | Bat coronavirus | China | NC_009657 |
